# Absence of Metalloprotease GP63 Alters the Protein Content of *Leishmania* Exosomes

**DOI:** 10.1371/journal.pone.0095007

**Published:** 2014-04-15

**Authors:** Kasra Hassani, Marina Tiemi Shio, Caroline Martel, Denis Faubert, Martin Olivier

**Affiliations:** 1 Department of Medicine, McGill University, Montréal, Québec, Canada; 2 Department of Microbiology and Immunology, McGill University, Montréal, Québec, Canada; 3 The Research Institute of the McGill University Health Center, Montréal, Québec, Canada; 4 Proteomics Discovery Platform, Institut de Recherches Cliniques de Montréal, Montréal, Québec, Canada; Institut national de la santé et de la recherche médicale - Institut Cochin, France

## Abstract

Protozoan parasites of *Leishmania* genus are able to successfully infect their host macrophage due to multiple virulence strategies that result in its deactivation. Recent studies suggest *Leishmania* GP63 to be a critical virulence factor in modulation of many macrophage molecules, including protein tyrosine phosphatases (PTPs) and transcription factors (TFs). Additionally, we and others recently reported that *Leishmania*-released exosomes can participate in pathogenesis. Exosomes are 40–100 nm vesicles that are freed by many eukaryotic cells. To better understand the GP63-dependent immune modulation of the macrophage by *Leishmania* parasites and their exosomes, we compared the immunomodulatory properties of *Leishmania major* (WT) and *L. major gp63^−/−^* (KO) as well as their exosomes *in vitro* and *in vivo.* Importantly, we observed that *Leishmania* exosomes can modulate macrophage PTPs and TFs in a GP63-dependent manner. In addition, our qRT-PCR analyses showed that WT parasites were able to downregulate multiple genes involved in the immune response, especially cytokines and pattern recognition receptors. KO parasites showed a strongly reduced modulatory capacity compared to WT parasites. Furthermore, comparison of WT versus KO exosomes also showed divergences in alteration of gene expression, especially of chemokine receptors. In parallel, studying the *in vivo* inflammatory recruitment using a murine air pouch model, we found that exosomes have stronger proinflammatory properties than parasites and preferentially induce the recruitment of neutrophils. Finally, comparative proteomics of WT and KO exosomes surprisingly revealed major differences in their protein content, suggesting a role for GP63 in *Leishmania* exosomal protein sorting. Collectively our data clearly establish the crucial role of GP63 in dampening the innate inflammatory response during early *Leishmania* infection, and also provides new insights in regard to the role and biology of exosomes in *Leishmania* host-parasite interactions.

## Introduction

Leishmaniasis is a spectrum of diseases caused by the protozoan parasites of genus *Leishmania*. This disease ranges from self-healing lesions of cutaneous leishmaniasis (caused mainly by *Leishmania mexicana* and *Leishmania major*) to potentially lethal visceral leishmaniasis (caused mainly by *Leishmania donovani* and *Leishmania infantum*). *Leishmania* parasites have a digenic life cycle. The elongated, motile and flagelated promastigote forms reside in the midgut of the female Phlebotomine sandfly, their invertebrate vector. When the sandfly takes a bloodmeal, the parasites are subcutaneously injected into the mammalian host. There, they are taken up by phagocytic cells and transform into amastigotes in the phagolysosome of the macrophages, their definitive mammalian host cell. Nonmotile and roundshaped amastigotes propagate in the phagolysosome, eventually leading to cell rupture and infection of adjacent cells. The cycle is completed when amastigotes and infected cells are picked up in another bloodmeal [1].

GP63 is a zinc-dependent metalloprotease that exists abundantly on the surface of promastigotes, attached via a GPI-anchor [2]. Cleavage of the GPI anchor via phospholipase C causes constant shedding of GP63 to the extracellular space. In addition, GP63 is also secreted directly from the parasite via the flagellar pocket. It has been shown that intracellular pools of GP63 exist that can be released upon certain extracellular triggers [3]. The genes encoding for this protease exist as a multigene array in the *Leishmania* genome. Different GP63 genes have subtle differences in sequence as well as expression pattern; however the exact differences among different GP63 genes are not fully characterized.

*Leishmania* parasites are able to successfully infect mammalian macrophages thanks to their multiple mechanisms of immune subversion and evasion [4]. We and others have reported that upon infection of the macrophage, multiple signaling proteins such as IRAK-1, JAK2 and MAP Kinases, transcription factors STAT-1, AP-1 and NF-κB, and also the translational protein mammalian/mechanistic target of rapamycin (mTOR) are altered [5]–[8]. Importantly, we showed that GP63, the major surface protease of *Leishmania* is a key player in many of the above-mentioned modulations. Gomez *et al.* demonstrated that GP63 is able to gain access to the macrophage cytoplasm and cause cleavage of prominent protein tyrosine phosphatases (PTPs) of the cell [9]. PTPs are general negative regulators of the signaling pathways; therefore, their activation together with alteration of other macrophage signaling molecules results in inhibition of inflammatory and leishmanicidal functions of the macrophage and persistence of the parasite infection. In addition, modulation of AP-1, NF-κB and mTOR was also shown to be GP63-dependent [7], [8], [10]. The mechanism by which GP63 gains access to the macrophage cytoplasm remains unknown. However, multiple mechanisms including possible delivery via exosomes have been proposed for this transfer [11].

Exosomes are 40–100 nm vesicles that are released from a wide variety of eukaryotic cells. These vesicles are released through pathways of un-conventional protein secretion and are enriched in certain membrane and cytosolic proteins as well as RNA. Exosomes and also other secreted vesicles have been shown to transfer signals to target cells and modulate their signaling [12], [13].

Recent work by us as well as Silverman and collaborators has suggested *Leishmania* exosomes to have macrophage immunomodulatory properties [14]–[16]. We observed that upon temperature switch from 25°C to 37°C, a condition mimicking parasite’s entry to the mammalian host, a very quick and dramatic increase in protein and exovesicle release occurs in *L. mexicana*. We saw that *Leishmania* exoproteome containing exosomes is able to modulate PTPs, TFs and macrophage functions such as nitric oxide (NO) production [14]. Work by Silverman and collaborators also supports that *Leishmania* exosomes modulate the innate immune system and functions such as IFN-γ-induced IL-8 and TNF-α in monocytes, as well as expression of co-stimulatory molecules by maturated dendritic cells [15], [16].

Having seen the importance of GP63 in modulation of the macrophage and possible immunomodulatory functions *Leishmania* exosomes, we decided to have a deeper look at GP63-mediated modulation of the macrophage by *Leishmania* parasites and their exosomes. For this purpose, we compared the immunomodulatory properties of wild type (WT) *L. major* with *gp63^−/−^* (KO) parasites as well as their exosomes. We observed that *Leishmania* exosomes are able to modulate macrophage PTPs and TFs in a GP63-dependent manner. In addition we compared WT and KO parasites and exosomes *in vitro* and *in vivo* using quantitative real time PCR (qRT-PCR) and also studying the inflammatory recruitment in the murine air pouch model. We observed that WT parasites and exosomes have a stronger potency in controlling the inflammation and immune modulation than KO parasites and exosomes. These results match with our hypothesis that *Leishmania* GP63 plays a key role in taming the inflammatory response during early infection. We also surprisingly found that in the absence of GP63, the protein content of exosomes is dramatically altered, importantly suggesting a novel role for GP63 in exosomal protein sorting.

## Materials and Methods

### Ethics Statement

Animal experiments were performed in accordance with the Canadian Council on Animal Care (CCAC) Guidelines, approved by the Institutional Animal Care and Use Committees at the McGill University under ethics protocol number 4859.

### Cell and Parasite Culture

The immortalized B10R bone-marrow derived macrophages were derived from B10A.Bcg^r^ mice and were cultured as described previously [17]. Briefly, cells were cultured in Dulbecco’s modified Eagle’s medium (DMEM) (Gibco-BRL) supplemented with 10% heat-inactivated fetal bovine serum (FBS), streptomycin (100 µg/ml), penicillin (100 U/ml), and 2 mM L-glutamine at 37°C and 5% CO_2_. *L. major* WT and KO parasites of strain Friedlin were cultured in Schneider’s Drosophila Medium (SDM) supplemented with 10% FBS at 25°C. *L. major* GP63-Rescue (RSC) parasites were cultured in SDM in the presence of 100 ng/ml of Geniticin.

### Collection and Purification of Exosomes from Parasite Conditioned Medium (CM)

Purification of exosomes was performed according to standard methods [18]. Day 7 CM of WT and KO *L. major* parasites was centrifugated at 4,000 g for 20 min to clear out parasites. Supernatant was then centrifugated at 10,000 g to clear debris. Alternatively, supernatant was filtered through 0.45 µm filters (Pall) to clear debris. Exosomes were pelleted by 1 h centrifugation at 100,000 g. Pelleted exosomes were then resuspended and washed in 20 mM HEPES buffer pH 7.5. Exosomes were overlayed on a gradient of 0 to 2 M sucrose and centrifugated overnight for further purification. Fractions corresponding to 1.1 to 1.3 M of sucrose were collected. Collected fractions were then sterile-filtered through a 0.22 µm filter (Pall) and pelleted by 1 h centrifugation at 100,000 g. Pelleted exosomes were resuspended in sterile HEPES buffer and kept at −80°C until use. Exosomes were dosed using microBCA protein dosing kit (Thermo).

### Transmission Electron Microscopy

Purified exosomes were coated on fomvar Carbon grids, fixed in 1% glutaraldehyde and stained with 1% uranyl acetate. Grids were then visualized using FEI Tecnai 12 120 kV. Images were taken using AMT XR-80C CCD Camera System.

### Gelatin Zymography

Samples were mixed with a loading dye without a reducing reagent and were loaded without boiling on a 10% SDS-PAGE gel containing 1.2 mg/ml of gelatin. Following SDS-PAGE, gels were incubated for 1 h in a buffer containing 50 mM Tris pH 7.4, 2.5% TritonX100, 5 mM CaCl_2_ and 1 µM ZnCl for 1 h at RT. Gels were then briefly rinsed in distilled water and incubated overnight in a buffer containing 50 mM Tris pH 7.4, 5 mM CaCl_2_ and 1 µM ZnCl at 37°C. Gels were then stained and briefly destained using Coomassie blue.

### *In vitro* Infection

B10R macrophages were left untreated, infected with stationary WT or KO parasites at 1∶20 ratio, stimulated with 100 ng/ml of LPS or treated with various concentrations of WT or KO exosomes for 3 h. Macrophages were then washed with PBS and lysed.

### Western Blot

Western blotting of cell lysates and exosomes were performed according to standard protocols. Proteins blotted to Hy-bond nylon members (Amersham) were detected by antibodies against SHP-1 (Abcam), PTP1B (Abcam), TCPTP (Abcam), GP63 (Robert McMaster, University of British Columbia, Canada), LACK (Jean-Claude Antoine Institute Pasteur, France, post-humous), HSP83 (Greg Matlashewski, McGill University, Canada), and tubulin (Abcam). Anti-mouse or anti-rabbit antibodies conjugated to horse-radish peroxidise (HRP) (Amersham) were used as secondary antibodies. Membranes were then visualized by ECL Western blotting detection system (Amersham).

### In-gel PTP Assay

In gel PTP assay was performed as described previously [19]. Briefly, poly (Glu-Tyr) substrate was radiophosphorylated with FER protein kinase and 150 µCi of [γ-32P] deoxyadenosine 5′-triphosphate and precipitated by TCA. Precipitated substrate was then filtered through a Sephadex column and then incorporated in an SDS-polyacrylamide gel at the concentration of 2×10^5^cpm/ml. Cell lysates were run on SDS-PAGE and then gels were incubated for 20 h in a fixative buffer containing 50 mM Tris-HCl (pH 8.0) and 20% isopropanol. Gels were then washed twice in 50 mM Tris-HCl (pH 8.0), 0.3% β-mercaptoethanol (β-ME) for 30 min each. Gels were put in 6 M guanidine hydrochloride and 1 mM EDTA denaturation solution for 1 h, and then washed twice in a renaturation buffer containing 50 mM Tris-HCl (pH 8.0), 1 mM EDTA, 0.3% β-ME and 0.04% Tween 20 for 1 h each. Final renaturation was done overnight. Gels were then dehydated with 40% ethanol and dried. Autoradiography on gels was performed using Kodak film. Clear bands were indicative of active PTPs.

### Electrophoretic Mobility Shift Assay (EMSA)

EMSA was performed as described previously [6]. Briefly, nuclear proteins were extracted using an isotonic and then a hypotonic buffer. Extracted nuclear proteins were incubated with radiolabelled consensus sequences of NF-κB (5′-AGTTGAGGGGACTTTCCCAGGC-3′), AP-1(5′-AGCTCGCGTGACTCAGCTG-3′) and SP-1 (5′-ATTCGATCGGGGCGGGGCGAGC-3′) (Santa Cruz) as non-specific control. Samples were run on a native 4% acrylamide gel. Following electrophoresis, gels were dried and autoradiography was performed using Kodak film.

### Quantitative Real-time PCR (qRT-PCR)

B10R macrophages were left untreated, stimulated with LPS, infected with *Leishmania* parasites or treated with 15 µg/ml of exosomes as described above, for 8 h. Cells were then washed with PBS and total RNA was extracted using TRIzol reagent (Invitrogen) according to manufacturer’s protocol. Clearance of possible genomic DNA contamination was performed using DNase I (Promega) according to manufacturer’s protocol. 1 µg of total RNA was used for cDNA preparation using reverse transcriptase enzyme Superscript III (Invitrogen) and random oligo-hexamers (Invitrogen). Samples were then treated with *Escherichia coli* RNase H (Invitrogen) for clearance of RNA-DNA helices. qRT-PCR was performed using Qiagen SABioscience RT^2^ profiler arrays in a Strategene mx3000 thermocycler according to SABiosciences protocol. Results were analyzed by ΔΔCt method. Gene Ontology (GO) terms were acquired from Uniprot database via STRAP [20].

### Murine Air Pouch Injection

Air pouches were made on the back of 6-weeks old female BALB/c mice (Purchased from Charles River laboratories) as described previously [21]. 3 ml of sterile air was injected into the back of the mice via a 27½G needles 7 and 3 days prior to experiment. 10^7^ stationary parasites, 25 µg of exosomes or 20 µg of LPS were diluted in 1 ml of endotoxin-free PBS (Wisent) and were injected into the back of the mice. 6 h following injection, mice were sacrificed and the content of the air pouch was washed with 5 ml of PBS. Differential cell counting was performed using cell cytospin. Statistical analyses of the results were performed using Graphpad Prism software 5.0. One-way ANOVA and Benferroni’s multiple comparison tests were used with p-value threshold <0.05 to indicate statistically significant differences.

### Pseudomigration and Protein Digestion with Trypsin

Proteins (15 µg) were loaded on an SDS-PAGE polyacrylamide gel containing 10% sucrose and run for 1 cm into the resolving gel. The in-gel digestion was performed as described previously [22]. The gel lane was excised into 3 bands and each band was cut in 1 mm^3^ pieces. Gel pieces were first washed with water for 5 min and then dehydrated with acetonitrile (ACN). Proteins were reduced by adding the reduction buffer (10 mM DTT, 100 mM ammonium bicarbonate) for 30 min at 40°C, and then alkylated by adding the alkylation buffer (55 mM iodoacetamide, 100 mM ammonium bicarbonate) for 20 min at 40°C. Gel pieces were dehydrated and washed at 40°C by adding ACN for 5 min before discarding all the reagents. Gel pieces were dried for 5 min at 40°C and then re-hydrated at 4°C for 40 min with the trypsin solution (6 ng/µl of trypsin sequencing grade from Promega, 25 mM ammonium bicarbonate). The concentration of trypsin was kept low to reduce signal suppression effects and background originating from autolysis products when performing LC-MS/MS analysis. Protein digestion was performed at 58°C for 1 h and stopped with 15 µl of 1% formic acid/2% ACN. Supernatant was transferred into a 96-well plate and peptides extraction was performed with two 30-min extraction steps at room temperature using the extraction buffer (1% formic acid/50% ACN). All peptide extracts were pooled into the 96-well plate and then completely dried in vacuum centrifuge. The plate was sealed and stored at −20°C until LC-MS/MS analysis.

### LC-MS/MS

Prior to LC-MS/MS, protein digests were re-solubilized under agitation for 15 min in 10 µL of 0.2% formic acid. Desalting/cleanup of the digests was performed by using C_18_ ZipTip pipette tips (Millipore, Billerica, MA). Elutes were dried down in vacuum centrifuge and then re-solubilized under agitation for 15 min in 10 µL of 2% ACN/1% formic acid. The LC column was a C18 reversed phase column packed with a high-pressure packing cell. A 75 µm i.d. Self-Pack PicoFrit fused silica capillary column (New Objective, Woburn, MA) of 15 cm long was packed with the C18 Jupiter 5 µm 300 Å reverse-phase material (Phenomenex, Torrance, CA). This column was installed on the Easy-nLC II system (Proxeon Biosystems, Odense, Denmark) and coupled to the LTQ Orbitrap Velos (ThermoFisher Scientific, Bremen, Germany) equipped with a Proxeon nanoelectrospray ion source. The buffers used for chromatography were 0.2% formic acid (buffer A) and 100% ACN/0.2% formic acid (buffer B). During the first 12 min, 5 µL of sample were loaded on column at a flow rate of 600 nL/min and, subsequently, the gradient went from 2–55% buffer B in 100 min at a flow rate of 250 nL/min followed by a rapid increase to 90% buffer B and then came back at 2% buffer B for 10 min at a flowrate of 600 nL/min. LC-MS/MS data acquisition was accomplished using a eleven scan event cycle comprised of a full scan MS for scan event 1 acquired in the Orbitrap. The mass resolution for MS was set to 60,000 (at m/z 400) and used to trigger the ten additional MS/MS events acquired in parallel in the linear ion trap for the top ten most intense ions. Mass over charge ratio range was from 380 to 2000 for MS scanning with a target value of 1,000,000 charges and from ∼1/3 of parent m/z ratio to 2000 for MS/MS scanning with a target value of 10,000 charges. The data dependent scan events used a maximum ion fill time of 100 ms and 1 microscan. Target ions already selected for MS/MS were dynamically excluded for 25 s. Nanospray and S-lens voltages were set to 0.9–1.8 kV and 50 V, respectively. Capillary temperature was set to 225°C. MS/MS conditions were: normalized collision energy, 35 V; activation q, 0.25; activation time, 10 ms.

### Database Searching and Protein Identification

Samples were analyzed using Mascot (Matrix Science, London, UK; version 2.3.02). Mascot was set up to search the NCBI Leishmania major database (17505 entries) assuming the digestion enzyme trypsin. Mascot was searched with a fragment ion mass tolerance of 0.60 Da and a parent ion tolerance of 10.0 PPM. Carbamidomethyl of cysteine was specified in Mascot as a fixed modification. Oxidation of methionine and phospho of serine, threonine and tyrosine were specified in Mascot as variable modifications. Scaffold (version Scaffold_4.3.0, Proteome Software Inc., Portland, OR) was used to validate MS/MS based peptide and protein identifications. Peptide identifications were accepted if they could be established at greater than 95.0% probability by the Peptide Prophet algorithm [23] with Scaffold delta-mass correction. Protein identifications were accepted if they could be established at greater than 95.0% probability and contained at least 2 identified peptides. Protein probabilities were assigned by the Protein Prophet algorithm [24]. Proteins that contained similar peptides and could not be differentiated based on MS/MS analysis alone were grouped to satisfy the principles of parsimony. False Discovery Rate (FDR) analyses were performed by X!Tandem and Mascot Percolator.

### Bioinformatic Analyses

Label-free normalization, quantification and comparison of proteins among WT, KO and RSC samples were performed using Scaffold software and by the Exponentially modified protein abundance index (emPAI) [25] **.** Prediction of transmembrane domains was done via TMHMM [26]. Prediction of signal peptides was done via SignalP version 3.0 [27]. GP63 putative cleavage sites were counted according to the cleavage site suggested by Bouvier et al. [28]. Briefly, all sequences were scanned for the residue sequence Polar-Hydrophobic-Basic-Basic ([TSYNQC][AIFLMWVP][RHL][RHL]). GO comparisons were performed via Panther (www.pantherdb.org) [29].

## Results

### Purification of Exosomes from WT and KO *Leishmania* CM

**Figure 1:**
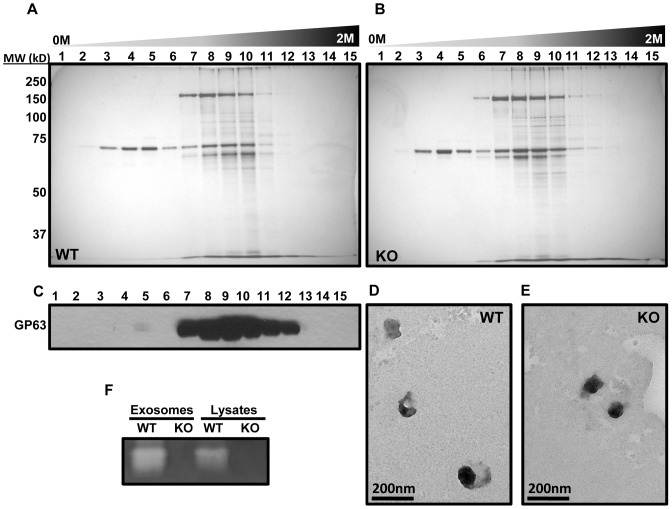
Purification of exosomes from CM of WT and KO parasites. A and B. Silver stainings of sucrose density gradient fractions of WT and KO exosomes respectively. Enrichment of bands can be observed in fractions 8, 9 and 10, which correspond to the density of exosomes. C. Western blotting of sucrose density gradient fractions of WT exosomes against GP63. Again accumulation of GP63 can be observed at fractions 8, 9 and 10. D and E. Transmission electron microscopy of WT and KO exosomes shows that WT and KO exosomes have similar size and morphology. Arrows point to exosomes. F. Gelatin zymogram of 3 µg of exosomes and lysates shows that GP63 remains active after exosome purification. All results are representatives of at least 3 independent experiments.

We extracted and purified exosomes from the CM of stationary WT and KO parasite cultures through multiple centrifugation, wash and filtration steps as detailed in the materials and methods section. We purified the exosomes from CM debris by density gradient centrifugation (Figure 1A, B and C). We picked fractions 8, 9 and 10 that correspond to the reported density of exosomes (∼1.2 M) and also showed highest enrichment of proteins and the surface molecule GP63 (Figure 1C). We did not observe any differences in the band patterns or density of exosomes acquired from WT and KO-derived exosomes (Figure 1A and B). We will call WT-derived and KO-derived exosomes as WT and KO exosomes for simplicity.

We evaluated the purity of the exosomes by transmission electron microscopy and observed that vesicles with the same size and morphology of exosomes have been purified from the CM of both WT and KO parasites (Figure 1D and E). We did not observe any difference in morphology of WT and KO exosomes. In addition, we did not observe any significant difference in exosome yield from parasite CM which was between 15 to 20 µg per 100 ml of parasite culture. Finally, using a gelatin zymography assay, we saw that GP63 remains active following the multiple steps of purification as well as freeze-thawing (Figure 1F).

### Modulation of Macrophage PTPs by *Leishmania* Exosomes

**Figure 2:**
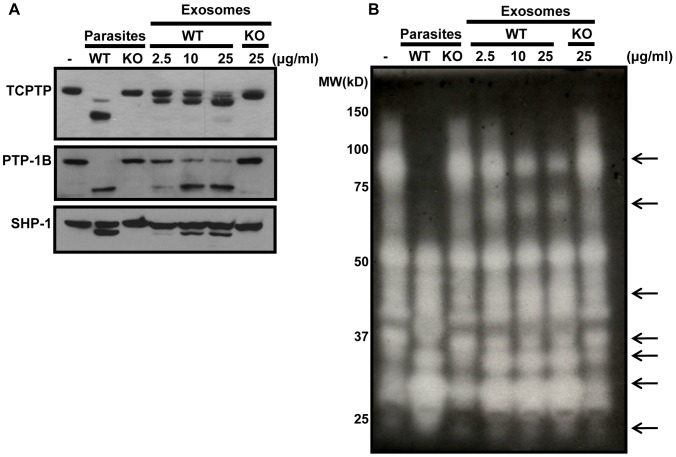
WT but not KO exosomes cleave macrophage PTPs dose-dependently. A. Western blotting shows that only WT but not KO parasites and exosomes can induce cleavage of prominent macrophage PTPs, TC-PTP, PTP-1B and SHP-1 after 3 h of incubation or infection. B. In gel PTP assay shows multiple modulations in the PTP profile of the macrophage following WT infection or incubation with WT exosomes, suggesting that cleavage fragments are enzymatically active. These modulations do not occur with KO parasites or with exosomes. All results are representatives of at least 3 independent experiments.

We previously showed that *Leishmania* as well as its exoproteome can cleave macrophage PTPs [14]. Since the exoproteome consists of both secreted exosomes as well as free GP63, we verified whether treatment with GP63 containing exosomes can induce cleavage of macrophage PTPs. We incubated increasing doses of *Leishmania* exosomes with B10R macrophages for 3 h. Our western blotting results show that as low as 2.5 µg/ml of WT exosomes is enough to cleave macrophage PTPs, SHP-1, PTP-1B and TCPTP in a similar fashion to infection with WT parasites, although to a lesser extent. We also observed that this ability increases dose-dependently (Figure 2A). However, we did not see any cleavage occurring after infection with KO parasites or incubation with KO exosomes. We further performed an in-gel PTP assay to look at the general profile of PTPs following treatment with exosomes. As can be seen in Figure 2B, WT exosome treatment results in a dose dependent modulation of multiple PTPs similar to WT infection. We saw no modulation following infection with KO parasites or KO exosome treatment. Together these results show that WT exosomes alone (but not KO exosomes) are able to induce cleavage and modulation of multiple PTPs similar to what we had observed with *L. mexicana* exoproteome [14].

### Differential Translocation of TFs by *Leishmania* Exosomes

**Figure 3:**
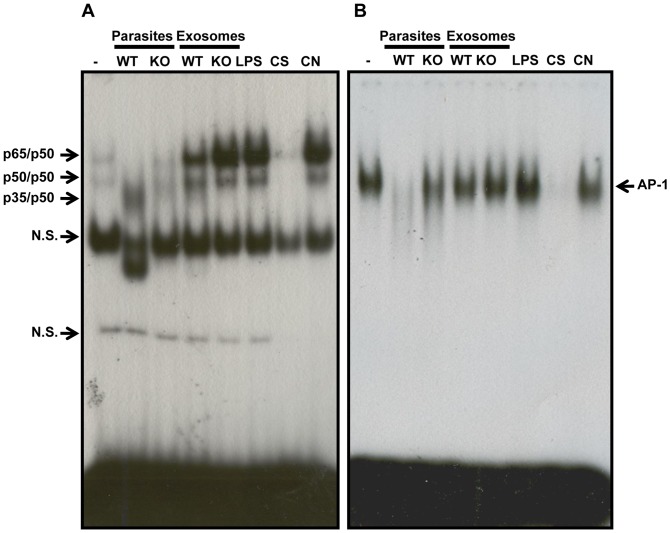
Modulation of TFs by *Leishmania* parasites and exosomes. EMSAs show that *Leishmania* infection can result in translocation of a modified form of NF-κB (A) and AP-1 (B) degradation as previously reported. Stimulation with parasite exosomes shows that KO exosomes induce stronger translocation of NF-κB and AP-1 into the nucleus and thus appear more inflammatory compared to WT exosomes. Results are representatives of at least 3 independent experiments. C.S. Specific Competitor, 100× concentration of non-labelled oligo. C.N. Non-Specific competitor, 100× concentration of non-labelled consensus SP-1 oligo. N.S. Non-specific.

We have previously shown that *Leishmania* infection strongly modulates macrophage TFs. Specifically, we reported that following *Leishmania* infection, AP-1 is fully degraded [8] and a modified form of NF-κB (named p35/p50) translocates into the nucleus [7]. We also showed that *Leishmania* exoproteome has similar effects on these TFs [14]. To compare the proinflammatory properties of *Leishmania* exosomes with and without GP63, we infected B10R macrophages with *Leishmania* parasites or treated them with 15 µg/ml of exosomes for 3 h. We looked at translocation of NF-κB and AP-1 by performing EMSAs on nuclear proteins (Figure 3). WT infection induced degradation of AP-1 and translocation of p35/p50 NF-κB into the nucleus. These effects were not observed after infection with KO parasites. However, stimulation with WT exosomes did not induce the p35/p50 form which shows that *L. major* exosomes and *L. mexicana* exoproteome have different immunomodulatory properties. Nevertheless, KO exosomes induced NF-κB translocation more strongly compared to WT exosomes (Figure 3A). As for AP-1, stimulation with WT but not KO exosomes induced a reduction in its levels compared to non-treated macrophages. In comparison, stimulation with KO exosomes induced a stronger translocation of AP-1 compared to WT exosomes, although this difference was not as pronounced as it was for NF-κB (Figure 3B).

### Modulation of Macrophage Gene Expression by *Leishmania*

Having observed differential translocation of TFs in response to WT or KO infection as well as treatment with WT and KO exosomes, we decided to look at the outcome of modulation of TFs in more detail by measuring gene expression. We used Qiagen-SABiosciences RT^2^ Profiler qRT-PCR arrays to measure expression of genes related to innate and adaptive immune response after 8 h of *Leishmania* infection or stimulation with exosomes. Of the 84 genes assayed, 60 genes (71%) were found to be at least 2-fold modulated compared to non-treated macrophages in at least one of the conditions (File S1). WT parasites induced the highest percentage of modulations (37 genes, 12 upregulated, 25 downregulated) while KO exosomes induced the lowest (19 genes, 16 upregulated, 3 downregulated). We interestingly observed a wide range of genes coding for secreted, surface and also cytoplasmic proteins to be differentially modulated by WT and KO parasites. Specifically, we observed induced downregulation or stronger downregulation of secreted proteins such as complement factor properdin (Cfp), interleukin 10 (Il10), TGF-β, (tgfb1), surface proteins such as CD14 surface antigen (CD14), Dectin-1 (Clec7a), Interferon gamma receptor 1, interleukin-1 receptor trype I (Il1r1), TLR8 (Tlr8), and Triggering receptor expressed on myeloid cells 1 (Trem1), and cytoplasmic proteins Lysozyme 1 (Lyz1), MAP-Kinase 8 or JNK1 (Mapk8), NF-κB inhibitor a (Nfkbia), Nod-like receptor CARD-like domain containing protein 4 (Nlrc4), Inducible nitric oxide synthase or iNOS (Nos2) in WT compared to KO parasites. In addition, we found Peptidoglycan recognition protein 1 (PGLYRP1), Conserved helix-loop-helix ubiquitous kinase (Chuck), Collectin sub-family member 12 (Clec12), CD55, Nos2, TLRs 1 and 8, Trem1, Mapk8, Nlrc4 and Il1r1 to be upregulated by KO parasites while downregulated by WT parasites (Summarized in Tables 1 and 2).

**Table 1:** List of genes that are downregulated by WT parasites measured by qRT-PCR.

#	Name	Description	WTP	KOP	WTX	KOX
**1**	Camp	Cathelicidin antimicrobial peptide	−7.11	−5.84	2.71	1.66
**2**	Cd14	CD14 antigen	−2.17	−1.22	2.04	3.03
**3**	Cd1d1	CD1d1 antigen	−2.08	−2.12	−1.30	−1.02
**4**	Cfp	Complement factor properdin	−5.41	−1.48	−1.59	−1.12
**5**	Clec7a	C-type lectin domain family 7, member a	−3.36	1.09	2.23	1.51
**6**	Cybb	Cytochrome b-245, beta polypeptide	−2.73	−2.51	2.23	2.27
**7**	Ifngr1	Interferon gamma receptor 1	−2.24	−1.87	1.30	1.69
**8**	Ifngr2	Interferon gamma receptor 2	−2.43	−2.11	−1.38	−1.06
**9**	Il10	Interleukin 10	−3.38	−1.78	−1.46	1.52
**10**	Il12rb2	Interleukin 12 receptor, beta 2	−2.99	−2.00	1.43	1.35
**11**	Il1b	Interleukin 1 beta	−11.79	−42.22	1.85	1.02
**12**	Il1r1	Interleukin 1 receptor, type I	−4.94	−1.93	1.30	−1.40
**13**	Irak1	Interleukin-1 receptor-associated kinase 1	−3.97	−3.15	−2.51	−1.51
**14**	Lyz1	Lysozyme 1	−4.41	−2.72	−1.02	−1.07
**15**	Mapk8	Mitogen-activated protein kinase 8	−2.94	−1.44	−1.06	−1.09
**16**	Nfkb2	NF-kappa B, p49/p100	−2.61	−4.64	−2.43	−1.76
**17**	Nfkbia	NF-kappa B inhibitor, alpha	−5.92	−3.06	1.16	−1.08
**18**	Nlrc4	NLR family, CARD domain containing 4	−3.02	1.19	1.15	−1.27
**19**	Nos2	Nitric oxide synthase 2, inducible	−3.94	2.73	−3.93	−1.49
**20**	Tlr2	Toll-like receptor 2	−5.26	−5.96	1.00	1.61
**21**	Tlr8	Toll-like receptor 8	−8.31	−3.96	−1.55	−1.40
**22**	Trem1	Triggering recept. expressed on myel. cells 1	−4.13	−1.05	11.71	4.89

Fold regulation against non-treated samples, calculated using ΔΔCt method, normalized against a panel of 4 house-keeping genes. Results are average of 2 replicates. WTP: WT parasites, KOP: KO parasites, WTX: WT exosomes, KOX: KO exosomes. Genes that are at least 2-fold downregulated by WTP are listed.

**Table 2:** List of genes upregulated by WT parasites or modulated by KO parasites.

#	Name	Description	WTP	KOP	WTX	KOX
**1**	Ccr3	Chemokine (C–C motif) receptor 3	1.33	2.29	1.22	3.82
**2**	Colec12	Collectin sub-family member 12	2.46	2.94	1.28	1.62
**3**	Hc	Hemolytic complement	17.69	2.01	1.00	1.00
**4**	Hmox1	Heme oxygenase (decycling) 1	4.21	4.13	1.95	2.16
**5**	Ifnb1	Interferon beta 1, fibroblast	2.22	4.21	1.10	1.43
**6**	Il1a	Interleukin 1 alpha	6.06	1.90	2.85	1.42
**7**	Il1f9	Interleukin 1 family, member 9	2.44	3.93	1.00	1.00
**8**	Il1rl2	Interleukin 1 receptor-like 2	1.73	2.39	1.75	3.86
**9**	Il1rn	Interleukin 1 receptor antagonist	12.64	11.79	3.43	3.01
**10**	Il6	Interleukin 6	3.36	1.69	2.89	1.00
**11**	Lalba	Lactalbumin, alpha	9.88	1.00	1.00	1.00
**12**	Lbp	LPS binding protein	6.06	−2.64	−1.38	−2.17
**13**	Ncf4	Neutrophil cytosolic factor 4	−1.06	−2.14	−1.02	−1.19
**14**	Ppbp	Pro-platelet basic protein	−1.75	−3.61	−1.04	1.17
**15**	Ptafr	Platelet-activating factor receptor	2.50	2.95	1.72	1.66
**16**	Tlr6	Toll-like receptor 6	2.79	1.44	1.11	1.27

Fold regulation against non-treated samples, calculated using ΔΔCt method, normalized against a panel of 4 house-keeping genes. Results are average of 2 replicates. WTP: WT parasites, KOP: KO parasites, WTX: WT exosomes, KOX: KO exosomes. Genes that are at least 2-fold upregulated by WTP or 2-fold up or down-regulated by KOP are listed.

WT and KO exosomes also showed divergence in induction of many genes, especially the cytokine receptors. Both chemokine receptors 3 and 4 (Ccr3 and Cxcr4) were upregulated by KO exosomes while remaining unchanged or downregulated by WT exosomes. We found 14 genes to be up or downregulated only by exosomes and not modulated by parasites (Summarized in Table 3).

**Table 3:** List of genes only modulated by exosomes.

#	Name	Description	WTP	KOP	WTX	KOX
**1**	Casp1	Caspase 1	−1.09	−1.15	2.20	1.66
**2**	Casp4	Caspase 4, apoptosis-related cysteine peptidase	−1.25	−1.08	2.11	2.91
**3**	Ccl2	Chemokine (C−C motif) ligand 2	1.07	1.99	3.27	3.32
**4**	Chuk	Conserved helix-loop-helix ubiquitous kinase	−1.37	1.63	1.47	2.47
**5**	Cxcr4	Chemokine (C-X-C motif) receptor 4	1.05	1.26	−2.57	2.37
**6**	Fn1	Fibronectin 1	1.46	−1.02	1.08	2.14
**7**	Ikbkb	Inhibitor of kappaB kinase beta	1.05	1.26	1.64	2.25
**8**	Il1f6	Interleukin 1 family, member 6	1.87	1.00	2.49	1.00
**9**	Il1r2	Interleukin 1 receptor, type II	−1.04	1.75	2.93	3.14
**10**	Mif	Macrophage migration inhibitory factor	1.18	1.31	2.31	1.62
**11**	Myd88	Myel. differentiat. primary response gene 88	−1.08	−1.08	5.35	−1.08
**12**	Tlr3	Toll-like receptor 3	1.04	1.82	4.06	2.44
**13**	Tnf	Tumor necrosis factor	−1.47	1.26	2.45	2.12

Fold regulation against non-treated samples, calculated using ΔΔCt method, normalized against a panel of 4 house-keeping genes. Results are average of 2 replicates. WTP: WT parasites, KOP: KO parasites, WTX: WT exosomes, KOX: KO exosomes. Genes that are at least 2-fold up or down regulated only by exosomes are listed.

Interestingly, we found that iNOS was downregulated by both WT parasites and exosomes, and not by KO parasites and exosomes; this strongly suggests a direct role for GP63 in modulation of iNOS expression. On the other hand, LPS binding protein (LBP) was downregulated by both KO parasites and exosomes and not by WT parasites and exosomes.

**Figure 4:**
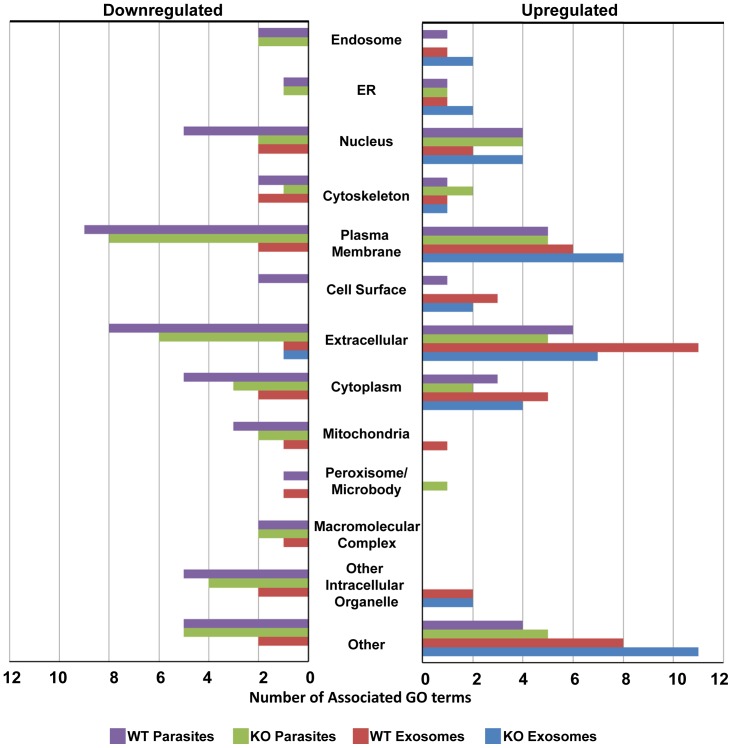
Gene ontology analyses of upregulated and downregulated genes. WT parasites, and to some extent KO parasites strongly downregulate expression of many immune-related genes, especially with plasma membrane, extracellular associated and nucleus GO terms, indicating secretory factors, receptors and transcription regulation proteins. Although WT exosomes do inhibit expression of certain genes, exosomes in general have a more stimulatory nature.

The differences in immune regulation ability of parasites and exosomes can also be seen when looking at Gene Ontology (GO) terms associated with up and downregulated genes (Figure 4). WT parasites (and to some extent KO parasites) downregulate genes with plasma membrane, extracellular associated and nucleus GO terms. These match with downregulation of different secretory factors, receptors and transcription regulation proteins as indicated above. On the other hand, exosomes show a more stimulatory profile, especially with plasma membrane and extracellular associated GO terms. Still, it can be seen that KO exosomes are more stimulatory compared with WT exosomes.

Overall, these results show that *Leishmania* infection results in downregulation of macrophage inflammatory gene expression in a GP63-dependent manner. Especially, detecting downregulation of many genes by WT parasites matches with our observation of GP63-mediated cleavage of pro-inflammatory TFs, AP-1 and NF-κB (Figure 3) by *Leishmania.* Our results also show that exosomes possess unique immunomodulatory properties.

### Measurement of the Inflammatory Response *in vivo*

**Figure 5:**
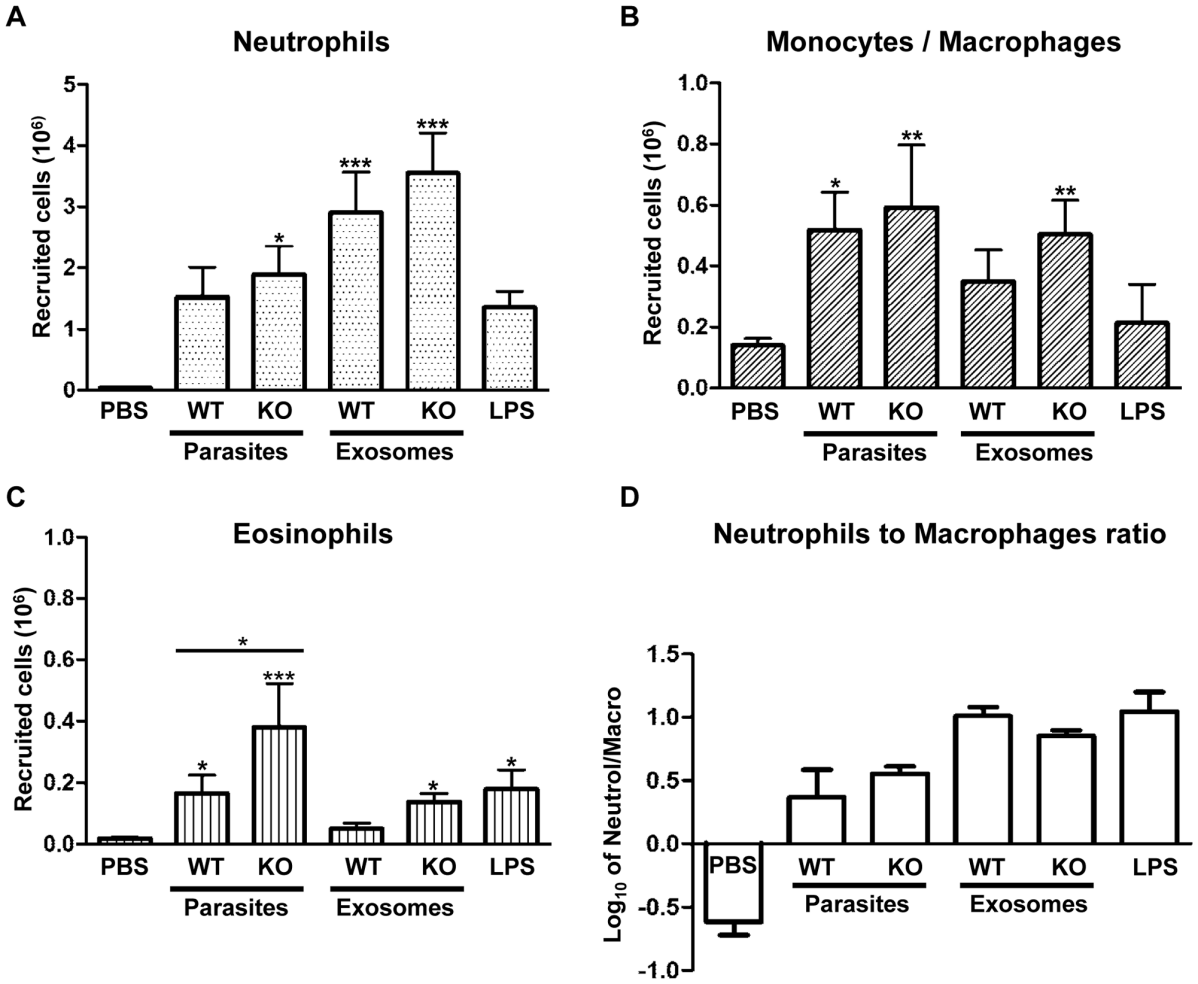
Recruitment of inflammatory cells to the air pouch in response to *Leishmania* parasites and exosomes. A, B and C show recruitment of neutrophils, monocytes/macrophages and eosinophils/mast cells calculated based on differential count respectively. D shows Log_10_ of the neutrophil/macrophage ratio, illustrating stronger recruitment of macrophages by parasites, compared to exosomes. Results show average of 3 separate experiments plus SEM. Statistical significance is measured against PBS-injected mice unless specified with a line (*: P-value <0.05, **: P-value <0.001, ***: P-value <0.0001).

We injected 10^7^ parasites or 25 µg of exosomes in a 1 ml volume into the air pouch of BALB/c mice and measured recruitment of inflammatory cells after 6 h. 1 ml of endotoxin-free PBS or 20 µg of LPS were injected as negative and positive controls respectively. Results indicate that both *Leishmania* parasites and exosomes induce recruitment of a mixed population of neutrophils and monocytes/macrophage with a smaller population of eosinophils/mast cells (Figure 5A–C). Exosomes induced recruitment of higher amounts of neutrophils, but less monocytes/macrophages and eosinophils/mast cells, compared to *Leishmania* parasites (Figure 5D). This indicates that exosomes are stronger inducers of the inflammatory response *in vivo*. On the other hand, the ability of parasites to recruit a larger percentage of macrophages can aid them in infecting more cells and better establishment of infection. Interestingly, we can observe a trend suggesting higher recruitment of inflammatory cells by KO parasites and exosomes. Although, this augmentation is not statistically significant, it matches with our findings on stronger proinflammatory properties of KO parasites and exosomes *in vitro*. Finally, recruitment of eosinophils is induced both by KO parasites and exosomes (Figure 5C). Interestingly, we observed by qRT-PCR that both KO parasites and exosomes induce expression of Ccr3 (Tables 1 and 3). Ccr3 is highly expressed on eosinophils and basophils and is an important chemokine receptor for their activation inflammatory recruitment [30], [31]. Overall, these results show that *Leishmania* parasites are able to induce recruitment of a mixed population of immune cells, especially monocytes/macrophages that could allow them in establishment of infection. Furthermore, we observe that *Leishmania* exosomes have stronger proinflammatory properties *in vivo*.

### Proteomic Analyses of Exosomes Suggest a Role for GP63 in Exosomal Protein Sorting

**Figure 6:**
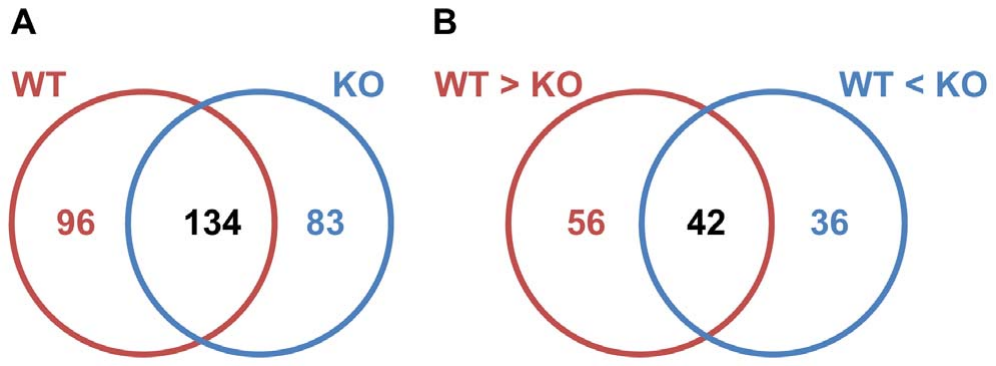
Proteomic analysis of WT and KO exosomes. A Venn diagram of proteins shared between WT and KO exosomes as well as unique proteins. B Venn diagram of common proteins between WT and KO exosomes, showing number of proteins with higher abundance (2×).

Having compared the ability of WT and KO parasites and exosomes in the modulation of macrophage signaling and functions in different levels, we performed mass spectrometry to compare the proteomic content of those exosomes and further identify their nature. Strikingly, we observed that a great amount of difference exists between the protein content of WT and KO exosomes: Of the total 313 proteins found with minimum 2 peptides in duplicate samples, only 134 were shared between WT and KO exosomes. 96 proteins were unique to WT exosomes while 83 proteins were unique to KO exosomes (Figure 6A, Files S2 and S3). Moreover, looking at the proteins shared between WT and KO exosomes, only 42 out of 134 had similar peptide counts. 56 proteins were 2 times or more abundant in WT, while 36 proteins were 2 times or more abundant in KO exosomes (Figure 6B).

Putting unique and modified proteins together, of the 230 proteins found in WT exosomes, 147 were WT only or had 2 times higher abundance in WT exosomes. On the other hand, of the 217 proteins found in KO exosomes, 119 were KO only or 2 times higher in abundance in KO exosomes.

**Figure 7:**
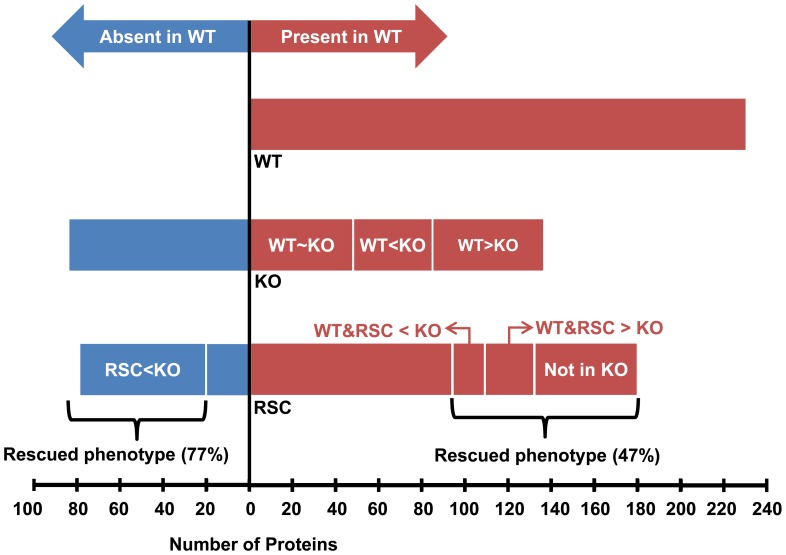
RSC exosomes show an intermediated phenotype between WT and KO in terms of presence and abundance of proteins. Proteins found in exosomes have been categorized as present (red) or absent (blue) in WT exosomes. Considering presence and absence of proteins, and also having similar abundance to WT, compared with KO, RSC exosomes show an intermediate phenotype between WT and KO exosomes. Refer to the results section for more detailed description.

To further verify that this effect is due to absence of GP63, we extracted and purified exosomes from CM of *L. major* GP63-Rescue parasites (RSC). RSC parasites are KO parasites that episomally express GP63 gene 1 as described previously [32]. RSC exosomes did not show any difference in density, morphology or yield from WT or KO exosomes (Data not shown). Mass spectrometry analysis of RSC exosomes showed an intermediate phenotype between WT and KO (Figure 7, Files S2 and S3).

RSC exosomes did not contain or had 2 times or lower abundance in about 77% of the proteins that were originally found only in KO exosomes (Figure 7, left side). Additionally, they did contain some proteins that were originally found only in WT exosomes and not in KO exosomes. Plus, abundance levels of some of the proteins found in RSC exosomes, matched those of WT and not KO (WT&RSC<KO, WT&RSC>KO; Figure 7, right side). Overall in RSC exosomes, 64% of proteins that had a difference in presence or abundance between WT and KO exosomes showed a return or a trend towards WT levels.

Proteins common between WT and KO exosomes included common proteins known to be enriched in exosomes, such as heat shock proteins. Interestingly, proteins that showed an increase in KO exosomes included a very high percentage of hypothetical proteins and many proteins with unknown functions (Table 4). Figure S1 compares GO terms associated with proteins enriched in WT or KO exosomes (high in WT or high in KO exosomes).

**Table 4:** Bioinformatic comparison of proteins enriched in WT, KO and RSC exosomes.

Property	WT>KO	RSC^1^	WT<KO	RSC^2^
**Number of proteins**	147	66 (45%)	119	78 (66%)
**Transmembrane** A	20 (14%)	15 (23%)	47 (39%)	38 (49%)
**GP63 Cut sites** B	537 (Ave. 3.6)	201 (Ave. 3.0)	254 (Ave 2.1)	164 (Ave. 2.1)
**Hypothetical**	45 (31%)	19 (29%)	58 (49%)	39 (50%)
**Signal peptide** C	10 (7%)	2 (2%)	10 (8%)	7 (9%)

Refer to the results section for detailed description.

1 Proteins whose phenotype was rescued in RSC exosomes: Present only in WT and RSC or higher than KO in WT and RSC.

2 Proteins whose phenotype was rescued in RSC exosomes: Absent in WT and RSC or lower than KO in WT and RSC.

A Transmembrane proteins predicted by TMHMM [26].

B Putative GP63 cutsites predicted according to [28].

C Presence of signal peptide predicted by SignalP [27].

Refer to text for detailed description.

Furthermore, bioinformatic analysis of the acquired proteins revealed interesting trends in the proteins that were modified in WT, KO and RSC exosomes. For simplicity of analyses, we merged proteins that were unique to WT exosomes and also had a higher abundance in WT exosomes compared to KO exosomes in one group of 147 proteins (Table 4, first column). Together, they account for proteins that are lost or reduced in the absence of GP63. On the other hand, proteins that were unique to KO exosomes (lost in WT) and had a higher abundance in KO exosomes, compared to WT exosomes were also merged to a group of 119 proteins (Table 4, third column). Together, they account for proteins that are gained or increased in the absence of GP63. Interestingly, there are high percentages of hypothetical and also transmembrane proteins in proteins that are gained or increased in KO exosomes. On the other hand, proteins lost or decreased in KO have a higher average of putative GP63 cut-sites (calculated from [28]). From the proteins lost or decreased in the absence of GP63, we saw 45% to show a return of phenotype (Presence in RSC, or higher abundance in KO). However, the percentage of proteins gained or increased in absence of GP63 that showed a return of phenotype was 66% (Columns 2 and 4 in Table 4). Overall, these account for a 54% return of WT phenotype in RSC exosomes, which is understandable, considering that RSC parasites only express GP63 gene 1 of the complete GP63 gene array.

**Figure 8:**
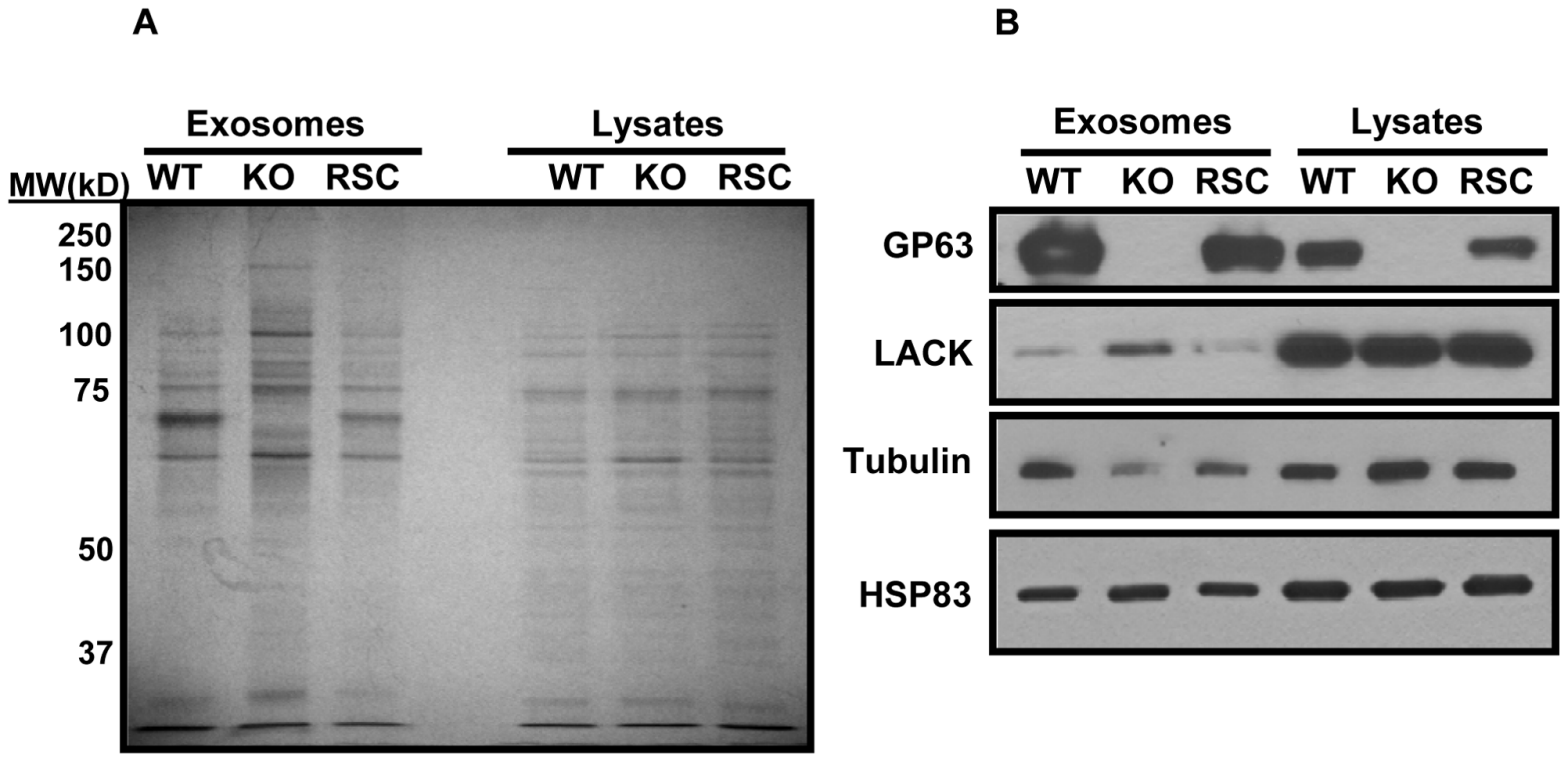
Verification of the proteomic data. A. Silver staining of 2 µg of exosomes and lysates shows that despite similarity of lysate band patterns, WT and RSC exosome band patterns differ from that of KO exosomes. B. Western blotting also shows equal levels of protein in parasite lysates, except for GP63 that is absent in KO. However, levels of tubulin and LACK protein appear to change in KO exosomes and go back to their WT levels in RSC. HSP83 levels appear to be unaffected. All results are representatives of at least 3 independent experiments.

Next, to verify the results obtained by mass spectrometry, we ran equal amounts of WT, KO and RSC exosomes as well as parasite lysates on SDS-PAGE and performed silver staining and western blotting (Figure 8). Silver staining of WT, KO and RSC exosomes clearly shows similar band patterns between WT and RSC, but different band patterns for KO exosomes (Figure 8A). Interestingly, the band patterns of WT, KO and RSC lysates appear to be very similar which suggests that the differences observed in the exosomes are not due to differential protein expression, but differential sorting of proteins into exosomes. This can be further observed by our western blotting results, where we show that abundance of LACK and tubulin is altered in KO exosomes; however, abundance of these proteins stays in similar levels in WT and RSC exosomes (Figure 7B). In addition, cellular levels of LACK and tubulin have remained unchanged among all three strains, again suggesting that the differences among exosomes are due to protein sorting and not differential gene expression.

Together, our data clearly suggest, for the first time, that GP63 could have a role in un-conventional secretion and protein sorting into the exosomes.

## Discussion

Research by several groups has now established GP63 as a critical virulence factor of *Leishmania*. This promiscuous protease is capable of interaction with various components of the immune system and their modulation in the parasite’s benefit [7]–[10], [32]–[36]. Here, we looked at the effect of GP63-mediated immune modulation on macrophage gene expression and *in vivo* pro-inflammatory recruitment. We further showed that GP63-bearing *Leishmania* exosomes have inflammatory properties and are also capable of macrophage immune modulation at both signaling and gene expression levels.

Our results evidently show that WT *Leishmania* parasites are more potent in control of inflammation compared to KO parasites. This is especially well demonstrated by our qRT-PCR results where we observed downregulation of many immune-related genes following infection with WT but not KO parasites (Tables 1 and 2 and Figure 4). In addition, besides corroborating and further extending what was already known about modulation of gene expression by *Leishmania*, such as downregulation of iNOS [37] and induction of chemokines [7], our results exhibit interesting clues on differential modulation of macrophage gene expression by WT and KO parasites. For instance, many genes downregulated after *Leishmania* infection code for different kinds of immune receptors: Adenosine receptor 2a, Interferon gamma receptor 2, Interleukin-12 receptor, TLRs 2 and 8. Moreover, many receptors and adaptors are downregulated after infection with WT parasites only: Dectin-1 (Clec7a), IL-1 receptor type I, Nlrc4 and Trem1. Downregulation of inflammatory receptors can be another mechanism that renders *Leishmania*-infected macrophages unresponsive to external stimulation, besides activation of PTPs and deactivation of signaling proteins. For many of these receptors, as well as other genes that we observed to be modulated after *Leishmania* infection, such as LPS-binding protein and Interleukin-1 receptor antagonist, there are currently no published reports of involvement in susceptibility or resistance to *Leishmania.* Thus, their role in the innate immune response to *Leishmania* as well as their possible application in therapeutics needs to be further dissected. Nevertheless, we were able to establish a link between GP63-mediated alteration of macrophage TFs, and inhibition of gene expression by *Leishmania.*

Another focus of this study was the comparison of the role of WT and KO exosomes in modulation of the inflammatory response. Exovesicle release appears to be universally conserved in life [38]. Secreted vesicles of pathogenic organisms are of great interest and importance, especially now that it is believed they could play roles in host-pathogen interactions [14], [16], [39]–[42]. We here saw that *Leishmania* exosomes share some immunomodulatory features with *Leishmania* parasites as well as *Leishmania* exoproteome. These included GP63-dependent cleavage of macrophage PTPs, reduction in translocation of pro-inflammatory TFs, and also reduced induction of proinflammatory gene expression compared to their KO counterparts. It is important to mention here that GP63 is present as a gene array of 7 in the *L. major* genome. These highly similar proteins have differences in sequence, regulation of expression, localization and biochemistry, which could lead to different pathogenic abilities [2], [3]. Especially, since most of GP63-mediated immune modulation occurs through entrance of the protease to the macrophage cytoplasm, it is possible that different forms of GP63, based on their sequence and structure, as well as presence or absence of a GPI-anchor, have different abilities to enter the cytoplasm or the nucleus, compared to one another. Therefore, it is conceivable that the proinflammatory properties of exosomes would not exactly follow those of the infecting parasite or the exoproteome, where a more complete cocktail of proteases is present. Santarem et al. have also recently shown that exoproteome and exosomes have expectedly different protein compositions [43]. Additionally, our previous study on *Leishmania* exoproteome used *L. mexicana* as a model [14]. Compared to *L. major*, *L. mexicana* is a more potent immunomodulatory parasite with more GP63 expression in addition to stronger secretory activity ( [2] and unpublished observations). Nevertheless, GP63-mediated modulation of macrophage PTPs and TFs by *Leishmania* exosomes, as well as modulation of certain inflammatory genes especially iNOS, Ccr3 and Cxcr4, show the abilities of these vesicles in pathogenesis. Generally, our results suggest that first; *Leishmania* exosomes possess immunomodulatory features that could aid the parasite in establishment of infection. Second, they show that presence of GP63 is important for the immunomodulatory features of *Leishmania* exosomes.

In addition to studying the *in vitro* inflammatory response to *Leishmania* parasites and exosomes from signaling and gene expression points of view, we also looked at the proinflammatory response *in vivo*. Previous reports have shown that a mixed population of monocytes and neutrophils arrives at the *L. major* infection site [21]. We observed that exosomes also recruit a mixed population of inflammatory cells, however consisting of more neutrophils than macrophages. This suggests that exosomes have a stronger pro-inflammatory nature compared to parasites, while *Leishmania* parasites are able to induce recruitment of a higher percentage of macrophages that could potentially get infected as their definitive host cells. Additionally, we observed an increase in eosinophil recruitment by KO parasites and exosomes. We believe that this increase could be possibly related to the increased expression of Ccr3 in response to KO parasite infection or exosome treatment, as we were able to detect it by qRT-PCR. However, it is beyond the scope of this study to speculate on the role these cells could play during the course of *Leishmania* infection. Finally, we could observe a trend, however not statistically significant, suggesting higher recruitment of inflammatory cells by KO parasites and exosomes, compared to their WT counterparts. While this trend matches our findings on the anti-inflammatory properties of GP63 *in vitro*, it also shows that more complicated mechanisms are involved during the *Leishmania*’s host-parasite interactions *in vivo*.

Finally, by performing comparative proteomic analyses of WT, KO and RSC exosomes, we were able to show for the first time that GP63 might play a role in exosomal protein sorting. Our results show that the majority of proteins found in WT exosomes are lost or decreased in abundance in KO exosomes, and vice versa. In addition, analyzing the proteomic content of RSC exosomes, we observed an intermediate phenotype further supporting the role of GP63 in this observed difference. RSC parasites only express GP63 gene 1 of the complete gene array [32] and thus it cannot be expected from them to show a complete return to the WT phenotype. To our knowledge, there are currently no reports of GP63’s involvement in any of the parasite’s physiological processes and the underlying reason for the stark difference between the exosomal content of WT and KO exosomes in unclear. However, looking closely at proteins modified in abundance among WT, KO and RSC exosomes, we were able to see trends such as increased or decreased frequency of transmembrane proteins, hypothetical proteins and putative GP63 cleavage sites (Summarized in table 4) which could provide clues on the mechanisms behind this phenomenon. For instance, it is possible that more transmembrane proteins find their way to the KO exosomes, because the exosome surface is not covered with GPI-anchored GP63. Also, since proteins enriched in WT exosomes have a higher average of putative GP63 cut-sites, it is possible that cleavage via GP63 would be required for sorting of certain proteins into exosomes.

Previous work by Silverman *et al.* also suggested that *L. donovani* exosomes are immunomodulatory to macrophages and DCs, thus aiding the parasite in establishment of infection [15], [16]. They also compared the immunomodulatory properties of wildtype vs. *HSP100^−/−^* exosomes showing loss of virulence in the latter [16]. HSPs have been found in almost all exosomes studied to date and it is not a surprise that deletion of an integral member of the exosome would result in extreme modulation of its content and loss of physiological function [12]. Interestingly, Silverman *et al*. also show that one of proteins lost in *HSP100^−/−^* exosomes is indeed GP63, whose roles in virulence are evident.

In conclusion, we showed that *Leishmania* GP63 allows for modulation of macrophage gene expression and the *in vivo* inflammatory response. We also showed that *L. major* KO exosomes are more pro-inflammatory than their WT counterparts. These differences further add to the possible roles of exosomes in immune modulation and aiding the parasite in establishment of infection. Finally, our proteomic data suggest a role for GP63 in exosomal protein sorting of *Leishmania.* Our findings provide a deeper understanding of *Leishmania* host-parasite interactions, by giving novel insights on GP63-mediated immune modulation. In addition, we present new putative targets for studying the innate immune response to *Leishmania* and development of therapeutics.

## Supporting Information

Figure S1 **Comparison of GO terms associated with proteins enriched in WT (red) or KO (blue) exosomes. GO terms were acquired via Panther.**
(PDF)

File S1 **Complete qRT-PCR data.**
(XLS)

File S2 **Peptide report, Spectrum report and Spectral count of LC/MS/MS analysis of WT, KO and RSC exosomes.**
(XLSX)

File S3 **Normalized emPAI values of WT, KO and RSC exosomes, and correlation coefficients.**
(XLSX)
