# Supplementary material for: Absence of Metalloprotease GP63 Alters the Protein Content of Leishmania Exosomes
Source: PLoS One. 2014 Apr 15;9(4):e95007. doi: 10.1371/journal.pone.0095007 (PMC3988155; doi:10.1371/journal.pone.0095007)
Supplement: Figure S1 — Comparison of GO terms associated with proteins enriched in WT (red) or KO (blue) exosomes. GO terms were acquired via Panther. (PDF) [file pone.0095007.s001.pdf]

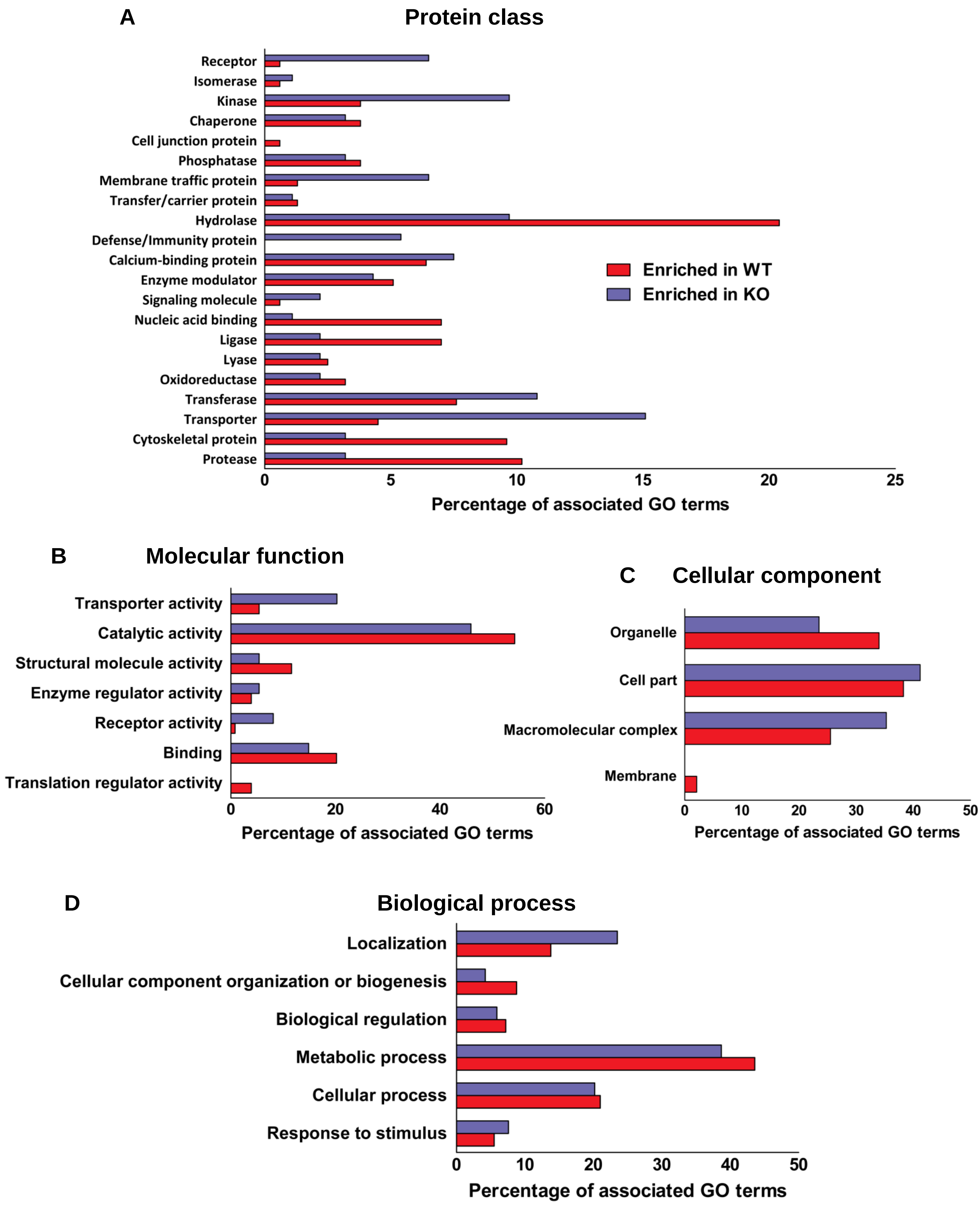

Supplemental Figure S1. Comparison of GO terms associated with proteins enriched in WT (red) or KO (blue) exosomes. GO terms were acquired via Panther.
